# Spontaneous Closure of a Post-biopsy Rectoprostatic Fistula via Dual Urinary Diversion: A Case Report and Review of Conservative Management

**DOI:** 10.7759/cureus.109821

**Published:** 2026-05-28

**Authors:** Ali Akjay, Najwa Jmil, Mourad Akdad, Jihad El Anzaoui

**Affiliations:** 1 Urology, Military Hospital Moulay Ismail, Meknes, MAR; 2 Department of Biology, Faculty of Sciences and Techniques, Moulay Ismail University, Errachidia, MAR; 3 Urology, Faculty of Medicine, Sidi Mohamed Ben Abdellah University, Fez, MAR

**Keywords:** conservative management, diabetes mellitus, prostatic abscess, rectoprostatic fistula, suprapubic catheterization, trus-guided biopsy

## Abstract

Rectoprostatic fistula (RPF) is an exceedingly rare but severe complication of transrectal ultrasound (TRUS)-guided prostate biopsy. It typically represents the terminal stage of a post-biopsy prostatic abscess that perforates the Denonvilliers’ fascia. We present a case of RPF successfully managed through a conservative “urinary-first” strategy. A 70-year-old male with type 2 diabetes mellitus and a glycated hemoglobin level of 8% presented eight weeks following a TRUS-guided biopsy with pathognomonic symptoms of fecaluria, pneumaturia, and urine passage per rectum. The patient had a history of recurrent *Enterococcus faecalis* urinary tract infections post-procedure. Digital rectal examination and multi-modal imaging (voiding cystourethrography, cystoscopy, and MRI) confirmed a Rivera class II rectourethral communication. Initial management with a transurethral Foley catheter was complicated by recurrent obstructions. A transition to a “dual-drainage” strategy, utilizing both a urethral catheter and a suprapubic tube (SPT), was implemented to ensure superior anatomical rest. After four weeks of diversion, follow-up cystoscopy and sigmoidoscopy confirmed complete spontaneous closure of the fistula. In high-risk patients, particularly those with diabetes, persistent post-biopsy symptoms should prompt immediate investigation for a prostatic abscess to prevent fistulization. This case highlights that a conservative approach using dual urinary drainage (SPT and Foley) can effectively facilitate secondary intention healing, potentially sparing patients from the morbidity of major reconstructive surgery.

## Introduction

Rectoprostatic fistula (RPF) is a rare pathological communication between the rectum and the prostate gland, most commonly occurring as a complication of surgical or interventional procedures. RPF is an exceptionally rare entity, with epidemiological data limited to isolated case reports. It is most commonly associated with a prostatic abscess or iatrogenic injury. Key risk factors include advanced diabetes, immunosuppression, and prior prostate instrumentation. It is important to distinguish RPF (involving the prostate) from rectourethral fistula, which directly communicates with the urethra and has different clinical and therapeutic implications. Despite its rarity, RPF carries significant morbidity and remains a diagnostic and therapeutic challenge.

Transrectal ultrasound (TRUS)-guided prostate biopsy remains a cornerstone of urological practice, widely used for the diagnosis of prostate cancer and for monitoring patients under active surveillance. Although generally considered safe, this procedure carries a risk of infectious complications, ranging from localized urinary tract infections to severe sepsis [1,2]. Among these complications, RPF represents an exceptionally rare but serious condition. It is most often the result of a post-biopsy prostatic abscess that progresses to perforation into the rectum due to ongoing inflammation and tissue necrosis [3-6].

Management of RPF ranges from conservative measures to complex surgical repair, with the choice depending on fistula characteristics, underlying etiology, and patient condition. To date, RPF following transrectal prostate biopsy has been only rarely described, and its optimal management remains undefined. Our case illustrates an unusual presentation and highlights the role of early imaging and minimally invasive management.

## Case presentation

A 70-year-old male with a history of type 2 diabetes mellitus and a glycated hemoglobin level of 8% presented with fecaluria, pneumaturia, and passage of urine per rectum, occurring eight weeks after undergoing a TRUS-guided prostate biopsy.

Eight days following the biopsy, the patient initially developed dysuria, chills, and fever (38°C). Urine culture identified *Enterococcus faecalis* as the causative organism. Laboratory findings showed leukocytosis (16,000/mm³) and a markedly elevated C-reactive protein level (200 mg/L), consistent with a significant infectious process, which was treated with ciprofloxacin, resulting in initial symptom resolution. However, six weeks after the procedure, he experienced recurrent dysuria and nocturia. A repeat urinary tract infection caused by *E. faecalis* was identified and managed with empirical antibiotics, leading to only transient improvement.

At approximately eight weeks post-biopsy, the patient reported passage of urine through both the rectum and urethra during micturition, with progressive worsening over the preceding two weeks. On digital rectal examination, a small indurated area was palpated approximately 4 cm from the anal verge, corresponding to Class II according to the Rivera classification. The prostate was firm and estimated to weigh approximately 55 g.

The diagnosis of rectourethral fistula was established using multimodal imaging, including voiding cystourethrography (VCUG), cystoscopy, and pelvic MRI. Retrograde cystourethrography demonstrated a well-opacified bladder with abnormal passage of contrast into the rectal lumen, confirming a fistulous communication between the lower urinary tract and the rectum. The tract appeared to originate from the prostatic urethra, with clear delineation of the rectal contour, and no evidence of vesical mass or obstruction.

On cystoscopic examination, an irregular mucosal defect was identified in the prostatic urethra, with surrounding erythematous and inflamed mucosa, without active bleeding or intraluminal tumor. These findings were highly suggestive of a communication between the prostatic urethra and the rectum.

Further evaluation with sagittal T2-weighted pelvic MRI revealed a well-defined hyperintense fistulous tract extending from the posterior aspect of the prostatic urethra to the anterior rectal wall, measuring approximately 44 mm in length and 4 mm in maximal diameter. The tract demonstrated fluid signal intensity with associated perilesional inflammatory changes, without evidence of suspicious mass or neoplastic infiltration. Collectively, these findings confirmed the diagnosis of rectourethral fistula.

The primary outcome was defined as complete fistula closure, assessed clinically by the resolution of urinary leakage per rectum and confirmed by the absence of a fistulous opening on cystoscopy and flexible sigmoidoscopy. Secondary outcomes included symptom resolution, catheter-related complications, and absence of recurrence during follow-up. Functional outcomes were systematically evaluated, including urinary continence status and the presence of lower urinary tract symptoms, to assess the overall success of management beyond anatomical healing.

Initial management consisted of urethral catheterization, successfully performed over a guidewire under flexible cystoscopic guidance, resulting in immediate symptomatic relief. However, over the following two weeks, the patient developed recurrent catheter obstruction associated with persistent urinary leakage per rectum.

Given the persistence of symptoms, a suprapubic catheter was placed under operative conditions. Dual urinary diversion via both the urethral catheter and suprapubic tube was maintained for four weeks, resulting in complete resolution of rectourethral leakage.

Follow-up evaluation with cystoscopy and flexible sigmoidoscopy, performed in collaboration with the colorectal surgery team, confirmed spontaneous closure of the rectourethral fistula (Figure 1). Both catheters were subsequently removed, and the patient remained asymptomatic with no evidence of recurrence. At the three-month follow-up, he continued to have no symptoms or evidence of fistula recurrence.

**Figure 1:**
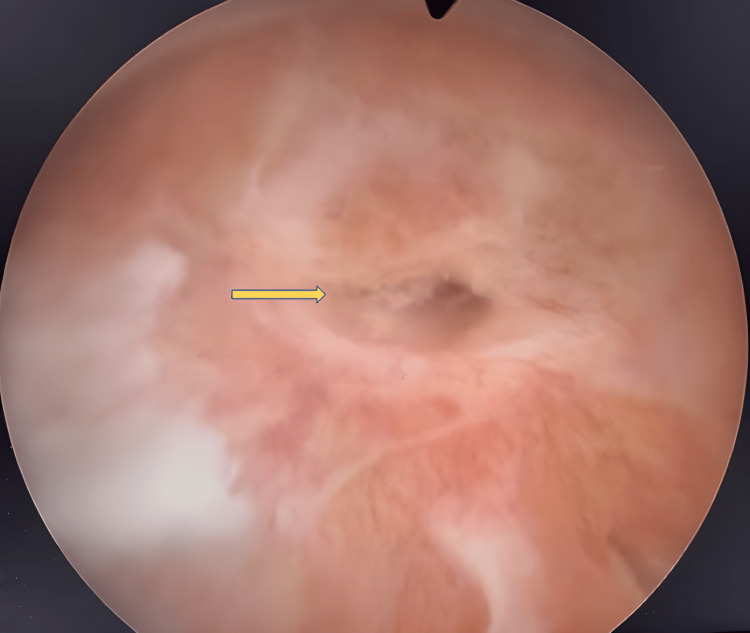
Cystoscopic findings of rectourethral fistula. Cystoscopic view demonstrating a mucosal defect in the prostatic urethra with a visible opening consistent with a rectourethral fistulous tract.

Cystoscopic view demonstrated a mucosal defect in the prostatic urethra with a visible opening consistent with a rectourethral fistulous tract (Figure 2).

**Figure 2:**
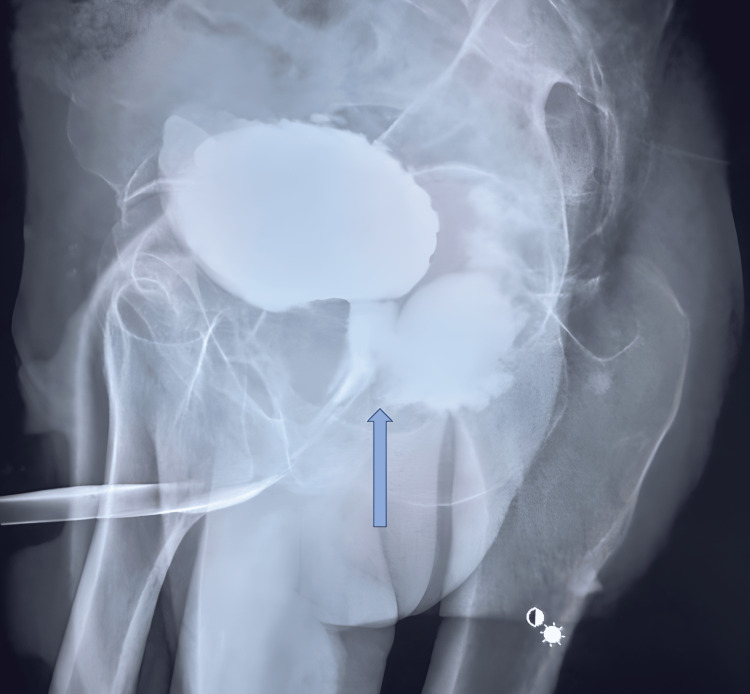
Voiding cystourethrogram demonstrating rectourethral fistula. Contrast-enhanced voiding cystourethrography showing opacification of the bladder with extravasation of contrast into the rectum, confirming the presence of a fistulous communication.

Contrast-enhanced VCUG showed opacification of the bladder with extravasation of contrast into the rectum, confirming the presence of a fistulous communication (Figure 3).

**Figure 3:**
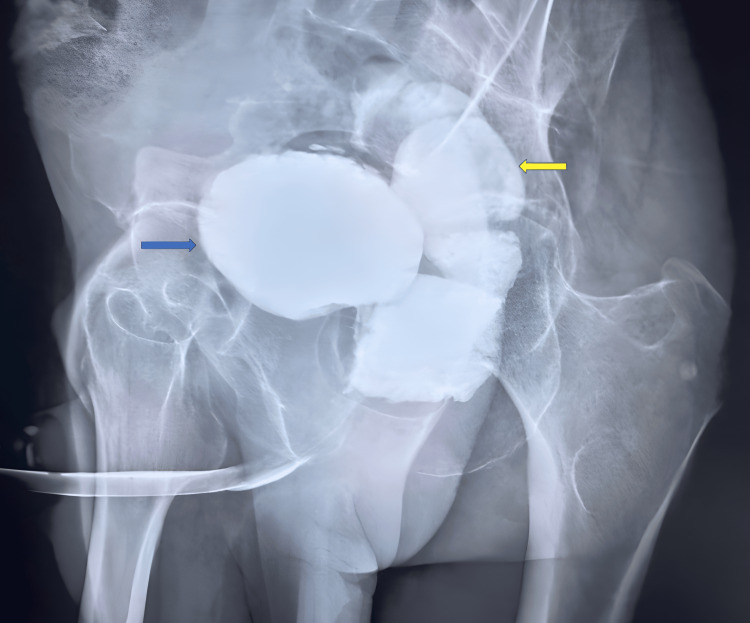
Persistent rectal opacification on voiding cystourethrography. Delayed imaging during voiding cystourethrography demonstrating continued filling of the rectal lumen from the urinary tract, further supporting the diagnosis of rectourethral fistula. Blue arrow: bladder. Yellow arrow: rectum.

Delayed imaging during VCUG demonstrated continued filling of the rectal lumen from the urinary tract, further supporting the diagnosis of rectourethral fistula. Sagittal T2-weighted pelvic MRI demonstrated a hyperintense tract extending between the prostatic urethra and rectum, consistent with a rectourethral fistula (Figure 4).

**Figure 4:**
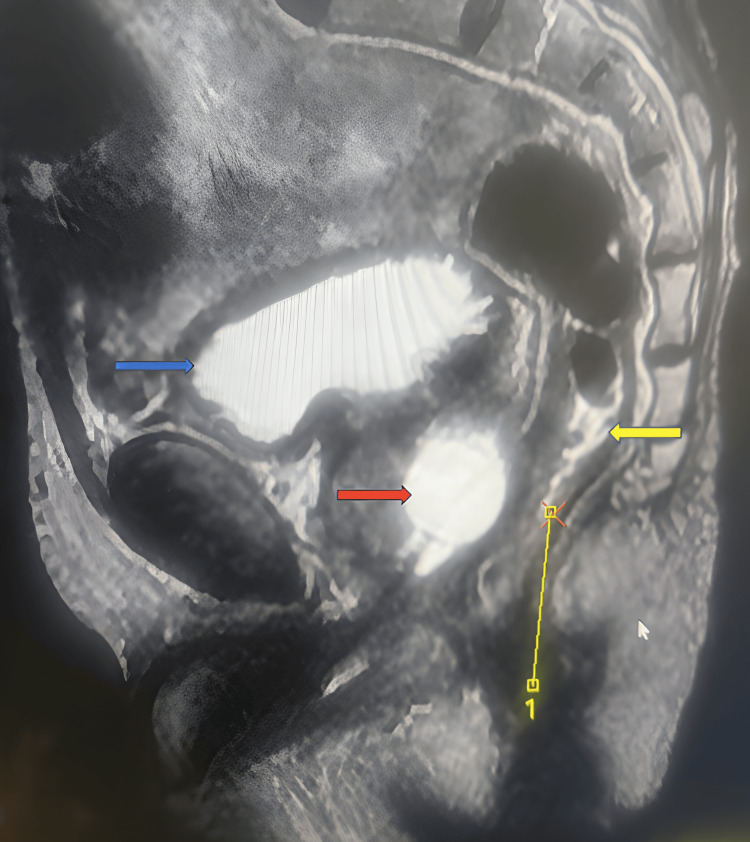
Pelvic MRI confirming fistulous tract. Sagittal T2-weighted pelvic MRI demonstrating a hyperintense tract extending between the prostatic urethra and rectum, consistent with a rectourethral fistula. Blue arrow: bladder. Yellow arrow: rectum. Red arrow: prostatic urethra.

## Discussion

RPF is an exceptionally rare but serious complication following TRUS-guided prostate biopsy. While post-biopsy infections are relatively common, progression to prostatic abscess occurs in fewer than 1% of cases [1,2], and fistulization into the rectum remains exceedingly uncommon, with only isolated cases reported in the literature.

The pathophysiology of RPF typically involves an ascending infection originating from rectal flora introduced during biopsy. This infection may evolve into a prostatic abscess, which, in the absence of timely drainage, can expand and erode adjacent structures. Due to the close anatomical relationship between the prostate and rectum, particularly at the level of Denonvilliers’ fascia, progressive inflammation and pressure necrosis may result in fistulous communication between the urinary and gastrointestinal tracts.

Several patient-related factors contribute to this progression. Among them, diabetes mellitus is a well-established risk factor, as impaired immune response and poor glycemic control facilitate the development of aggressive infections and abscess formation [1,5]. Additionally, prior urinary instrumentation and immunosuppression have been associated with an increased risk of complicated post-biopsy infections [2].

Clinically, the diagnosis should be suspected in patients presenting with pneumaturia, fecaluria, or urine passage per rectum, which are considered pathognomonic signs. Imaging plays a crucial role in confirming the diagnosis and defining the extent of the fistula. While TRUS and CT may aid in identifying associated abscesses, MRI remains the gold standard for precise delineation of the fistulous tract and surrounding anatomy [7]. VCUG provides dynamic confirmation of communication between the urinary tract and rectum.

Management of RPF remains challenging due to the lack of standardized guidelines, and treatment strategies must be individualized. Traditionally, surgical repair, including transrectal approaches (York-Mason), gracilis muscle interposition, or, more recently, robotic-assisted techniques, has been considered the definitive treatment, particularly for large or persistent fistulas. However, in selected cases, conservative management may be successful, especially for small, non-epithelialized fistulas of inflammatory origin. This approach is based on the principle of “anatomical rest,” achieved through adequate urinary and, in some cases, fecal diversion.

In this context, our approach differs in its use of dual urinary diversion as a primary strategy. While single catheterization has been reported in conservative management, it may be insufficient to ensure continuous low-pressure drainage, particularly in the setting of catheter obstruction or persistent leakage. Dual diversion, combining transurethral and suprapubic drainage, may offer more effective urinary decompression and promote “anatomical rest,” a principle recognized in fistula healing.

Compared with fecal diversion, which is often reserved for complex or contaminated cases, our approach avoids additional surgical morbidity. Similarly, unlike immediate surgical repair, which remains the standard for refractory cases, dual diversion represents a less invasive alternative that may be considered in carefully selected patients. The successful spontaneous closure observed in our case after four weeks supports this strategy, although evidence remains limited and further studies are needed to confirm its role.

## Conclusions

RPF is a rare but potentially preventable complication of TRUS-guided prostate biopsy. Early recognition of post-biopsy infections, particularly in high-risk patients such as those with diabetes mellitus, is essential to prevent progression to abscess and fistula formation. This case suggests that conservative management may be a viable option for RPF in carefully selected patients, although further studies are needed to confirm its effectiveness.
